# Contrasting Diversity Values: Statistical Inferences Based on Overlapping Confidence Intervals

**DOI:** 10.1371/journal.pone.0056794

**Published:** 2013-02-20

**Authors:** Ian MacGregor-Fors, Mark E. Payton

**Affiliations:** 1 Red de Ambiente y Sustentabilidad, Instituto de Ecología, A.C., Xalapa, Veracruz, México; 2 Department of Statistics, Oklahoma State University, Stillwater, Oklahoma, United States of America; University of Glasgow, United Kingdom

## Abstract

Ecologists often contrast diversity (species richness and abundances) using tests for comparing means or indices. However, many popular software applications do not support performing standard inferential statistics for estimates of species richness and/or density. In this study we simulated the behavior of asymmetric log-normal confidence intervals and determined an interval level that mimics statistical tests with P(α) = 0.05 when confidence intervals from two distributions do not overlap. Our results show that 84% confidence intervals robustly mimic 0.05 statistical tests for asymmetric confidence intervals, as has been demonstrated for symmetric ones in the past. Finally, we provide detailed user-guides for calculating 84% confidence intervals in two of the most robust and highly-used freeware related to diversity measurements for wildlife (i.e., *EstimateS*, *Distance*).

## Introduction

Measuring biodiversity is one of the major goals of ecologists around the world [1]. As suggested by Hubbell [2], biodiversity can be summarized by the species richness and relative abundances of a community in a given space and time. For decades, ecologists have used many different methods to calculate and contrast species richness, relative abundances, and/or diversity values. Most simply, ecologists often contrast species richness and abundances relative to sampling effort among different conditions using tests for comparing means (e.g., ANOVA, Kruskal-Wallis; [3]–[5]). Also, many indices have been developed to measure species richness and diversity (see Moreno [1], [6]–[8] for further details). However, many popular software applications do not support performing standard inferential statistics for estimates of diversity (e.g., species richness, density).

Recently, the use of two methods for quantifying species richness and individual densities have became very popular due to their robustness: (1) rarefaction curves produced by randomly re-sampling the pool of total individuals or sampling units, plotting the estimated number of species in relation to a given number of individuals or sampling units [9]–[11], and (2) distance-sampling calculation of densities (number of individuals per area unit - e.g., hectares, square kilometers), calculated based on the probability of detection of individuals at increasing distances from the observer and the size of the successfully surveyed area [8]. Both methods can be calculated using freeware. Rarefaction curves can be generated using the output from the software *EstimateS* [12], which computes the expected number of species as a function number of accumulated samples (sample-based rarefaction, denoted Sobs [Mao Tao] in *EstimateS*) with symmetric 95% confidence intervals (Sobs 95% CI Upper and Lower Bounds). Densities can be calculated using the software *Distance* [13], for which asymmetric 95% confidence intervals, based on assuming the distributions of the density estimate is log-normal, are output as a default by the program.

As software programs such as *EstimateS* and *Distance* output results that cannot be contrasted directly though inferential statistics, degree of overlap between confidence intervals has been proposed to assess statistical differences [14]. Such comparisons allow testing null hypotheses regarding different environmental conditions (e.g., habitats, treatments). Although other approaches to hypothesis testing for *Distance* have been shown to contrast density values effectively (e.g., ANOVA, *t*-tests), they often require experience using sophisticated processes in statistical packages.

**Figure 1:**
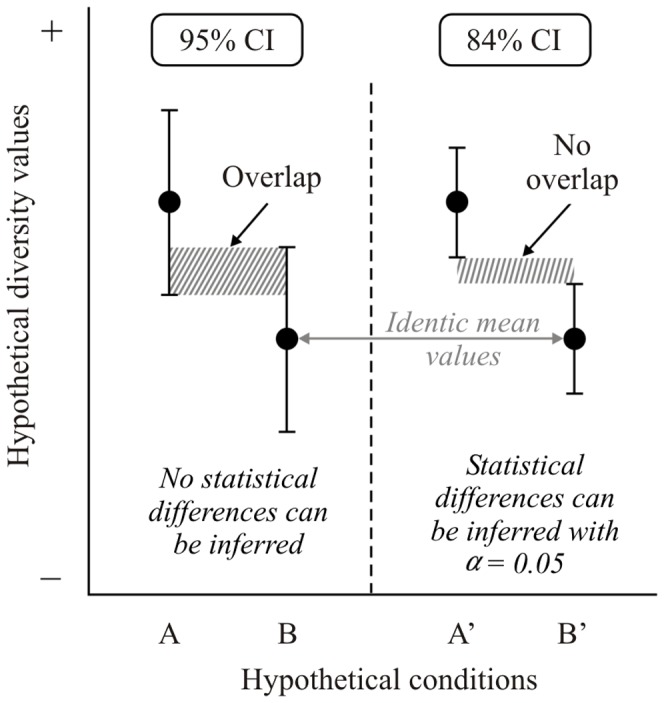
Hypothetical scenario comparing diversity values with 95% and 84% confidence intervals. In the example on the left (A vs. B), with 95% confidence intervals, no conclusion can be drawn regarding statistical difference in diversity values at P = 0.05. In the example on the right (A′ vs B′), with 84% confidence intervals but the same means as on the left, we can confidently infer that diversity values differ at P<0.05.

As demonstrated by Payton *et al.* [14], when comparing overlapping 95% confidence intervals of independent treatments with similar standard errors, non-overlapping confidence intervals represent significant differences in expectations with extremely low probabilities of Type I error (α <0.01), while no statistical inferences can be drawn with certainty if confidence intervals overlap but are not coincident. However, Payton *et al.* [14] showed that comparing 83–84% confidence intervals, instead of 95%, represents statistical tests with an α level of 0.05 (Fig. 1), the conventional criterion of significance for biological and ecological analyses [15].

As the 83–84% rule has previously been demonstrated only for normally distributed confidence intervals, in this study we simulated how asymmetric log-normal confidence intervals behave and determined a confidence interval level for mimicking two-sample statistical tests with α = 0.05. As the log-normal distribution is a normal distribution on the log-scale, we predicted that the 83–84% rule should also apply to asymmetric log-normal confidence intervals. We also describe how to calculate different percentage confidence intervals for rarefaction curves and distance-sampling based densities and indicate how to contrast them, representing a novel way to statistically compare species richness and density values robustly.

## Materials and Methods

### Simulations for Mimicking Pairwise Tests Based on Asymmetric Confidence Intervals

We performed simulations to establish the confidence intervals at which P<0.01, 0.05, and 0.10 Type I error was achieved, mimicking pairwise tests with PC SAS [16]. In order to explore how the proposed method behaves for various types of log-normal distributions, we created several combinations of the two parameters of the log-normal distribution (µ and σ). Specifically, we created 48 different log-normal distributions by utilizing 6 different levels for μ and 8 different levels for σ in an effort to cover a variety of different distributions. For the purposes of these simulations, we generated samples from parent populations which were generated by assuming different means and corresponding standard errors, which are functions of the parameters utilized to create these parent populations. Thus, as we assessed the behavior of asymmetric confidence intervals, we calculated a confidence interval for each of two samples drawn from the same population, each with alpha values varying from 0.05 to 0.25, at 0.01 increments. We calculated 10,000 iterations of each simulation scenario, including populations with different means extracted from the same parent populations. For each iteration, we calculated 0.75% to 0.95% confidence intervals in 1% increments, and we used this series of confidence intervals to determine the proportion of times the simulated confidence intervals overlap for each nominal level of confidence. Note that the log-normal distribution’s coefficient of variation is a function of σ only [17], so changing the mean of the distribution changes, by definition, the variance also.

## Results

For almost all of the scenarios contrasting samples with different means, the 84% confidence intervals provided overlap probability that best mimicked a two-tailed two population test with a 0.05 error rate. To mimic a 0.01 test, 94% confidence intervals would appear to be the proper choice. Confidence intervals at the 76% level best mimic a test with a 0.10 error rate (Tables 1, 2).

**Table 1:** Simulation results of 10,000 iterations calculating the overlap of confidence intervals of various sizes generated from log-normal populations with mean of 12.2 and variance of 0.08 (log-normal parameter values of μ = 2.5 and σ^2^ = 0.0005).

Size of CIs (%)	Average overlap	Size of CIs (%)	Average overlap
95	0.9946	84*	0.9543*
94	0.9898	83	0.9494
93	0.9899	82	0.9448
92	0.9855	81	0.9312
91	0.9838	80	0.9318
90	0.9802	79	0.9276
89	0.9768	78	0.9149
88	0.9687	77	0.909
87	0.9671	76*	0.9026*
86	0.9624	75	0.8966
85	0.9608		

Values that represent the preferred choice of confidence interval to mimic tests with alpha of 0.05 and 0.10 are marked with an asterisk (*). This table represents only one set of parameter values considered (among 48 sets considered) and is meant to represent typical results associated with the other population values considered in our simulations.

**Table 2:** Appropriate sizes of confidence intervals to simulate P = 0.05 and P = 0.10 size tests for various combinations of log-normal parameter values and associated means and variances.

μ	σ 2	Mean	Variance	Size of CIs (%)	Average overlap
4.5	0.0005	90.04	4.05	84	0.9541
				76	0.9032
	0.001	90.06	8.12	84	0.9546
				76	0.903
	0.0015	90.08	12.18	84	0.9507
				76	0.9049
	0.002	90.11	16.25	84	0.953
				76	0.9036
	0.0025	90.13	20.33	83	0.9506
				76	0.9065
	0.003	90.15	24.41	84	0.9541
				75	0.9001
	0.0035	90.17	28.51	84	0.9566
				77	0.9114
	0.004	90.2	32.61	83	0.9505
				76	0.9042
5.5	0.0005	244.75	29.96	84	0.952
				77	0.9071
	0.001	244.81	59.96	83	0.9517
				76	0.9043
	0.0015	244.88	90.01	84	0.9554
				75	0.904
	0.002	244.94	120.11	84	0.9509
				76	0.9049
	0.0025	245	150.25	85	0.9582
				76	0.901
	0.003	245.06	180.43	84	0.9534
				76	0.9022
	0.0035	245.12	210.66	84	0.9525
				76	0.9057
	0.004	245.18	240.94	83	0.9514
				76	0.9041
6.5	0.0005	665.31	221.37	84	0.9533
				76	0.9039
	0.001	665.47	443.08	84	0.9535
				76	0.9011
	0.0015	665.64	665.12	84	0.9557
				75	0.9017
	0.002	665.81	887.49	84	0.9524
				76	0.9012
	0.0025	665.97	1110.19	84	0.9517
				76	0.901
	0.003	666.14	1333.23	83	0.9508
				77	0.9091
	0.0035	66.31	1556.6	83	0.9504
				76	0.9042
	0.004	666.47	1780.3	84	0.9521
				76	0.9028

Results from simulations including 10,000 iterations.

## Discussion

**Figure 2:**
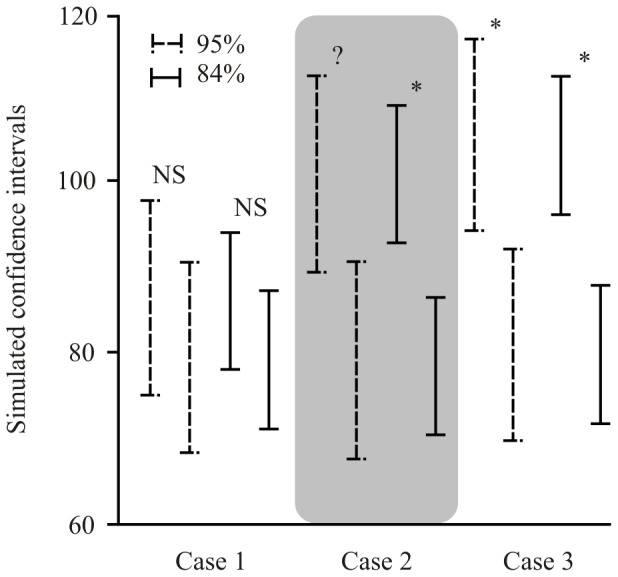
Comparison of the use of 95% and 84% confidence intervals in three replicates of our simulations. For this representative example, the data were created from a log-normal population with a mean of 90.2 and variance of 32.6. In case 1, the both sets of intervals overlap, both suggesting that no significant (NS) differences exist. Note, however, that the 95% confidence intervals will yield an error rate of less than 1%, while the 84% confidence intervals better mimic a 0.05 level test. In case 2, 95% confidence intervals slightly overlap, while 84% ones do not. For this situation, these two approaches would lead to different conclusions: (a) significant differences (*) when considering 84% confidence intervals, and (b) no statistical differences can be inferred using 95% confidence intervals (?). In case 3, none of the sets of intervals overlap, both suggesting that significant differences exist. Note, however, that statistical differences using 95% confidence intervals are assumed with an error rate of less than 1%, while that of 84% confidence intervals better mimic a 0.05 level test.

As predicted, our results show that comparing the overlap, or lack of it, between 84% asymmetric confidence intervals pertaining to different means mimics 0.05 tests surprisingly well (Fig. 1, 2). Thus, this study provides empirical evidence that the 84% rule is suitable for mimicking 0.05 statistical tests for both symmetric and asymmetric confidence intervals. However, we did not explore the statistical power of the method (regarding Type II errors), since the primary concern of this paper was to create a process that best mimicked an alpha-level test, and the use of overlapping 84% confidence intervals for this method would be more powerful, by definition, than using 95% intervals. Assessing power for this situation would involve constructing distributions with different means (and, by virtue of the nature of the log-normal distribution, different variances) and assessing the ability of the method to detect differences in overlapping confidence intervals with different means. Though our results have been demonstrated effective only for normal symmetric intervals and for log-normal asymmetric intervals, we believe that the 84% rule for mimicking 0.05 tests with overlapping confidence intervals might work effectively for other distributions. For example, comparing 84% confidence intervals for species estimation comparisons using widely used non-parametric estimators (e.g., Chao1, Chao2, ICE, ACE, Jackknife, Bootstrap), could mimic 0.05 tests. However, it remains to be tested.

In order to generate 84% confidence intervals for rarefaction analyses, the standard deviation of the observed species (Mao Tao SD) from the output file from *EstimateS* is needed. As standard deviations equal standard errors in *EstimateS* because infinite degrees of freedom are assumed in the calculation of Mao Tao SD, the latter must be multiplied by 1.372, the quantile (normal curve *z*-score) corresponding to two-sided intervals of 84% probabilities, with alpha = 0.16, and cumulative probabilities of 0.08 and 0.92. For example, if Mao Tao SD = 5.55, for example, 84% confidence intervals for that specific value of Mao Tao SD, which can vary in relation to the number of accumulated computed individuals in a rarefaction plot, are equal to the average value ±7.61.

As the *Distance* program can calculate user-selected levels for confidence intervals (default = 0.95) for distance-sampling density calculations, setting the confidence interval limits solves the issue. To accomplish this, go the “Analyses” button on the toolbar, select “Analysis details” and a new window will appear. Finally, select the “Misc” tab and modify the default value for confidence intervals (i.e., 95) to 84. Results output from the *Distance* program will now include 84% confidence intervals.

Wildlife species richness and density measurements of ecosystems are imperative in order to concentrate conservation actions in highly biodiverse areas [1]. In this paper, we demonstrated that the 84% rule mimics 0.05 pairwise statistical tests for both symmetric and asymmetric confidence intervals, with detailed users’ guides for calculating 84% confidence intervals in two of the most robust and highly-used freeware applications related to biodiversity (i.e., *EstimateS*, *Distance*). Thus, we encourage ecologists to use these programs to calculate species richness and individual density statistical expectations, applying this easy-to-use overlapping confidence interval method when making statistical inferences, which represents an alternative to the use of diversity indices.

## References

[1] Magurran AE (2004) Measuring Biological Diversity. Oxford: Blackwell Publishing.

[2] Hubbell SP (2001) The Unified Neutral Theory of Biodiversity and Biogeography. Princeton: Princeton University Press.

[3] Fernández-Juricic E (2001) Avian spatial segregation at edges and interiors of urban parks in Madrid, Spain. Biodivers Conserv 10: 13031–316.

[4] Gaines WL, Haggard M, Lehmkuhl JF, Lyons A L, Harrod RJ (2007) Short-term response of land birds to Ponderosa Pine restoration. Rest Ecol 15: 670–678.

[5] Moreno C, Rodríguez P (2010) A consistent terminology for quantifying species diversity? Oecologia 163: 279–282. 20213149 10.1007/s00442-010-1591-7

[6] Moreno CE (2001) Métodos para Medir la Biodiversidad. Zaragoza: M&T-Manuales y Tesis SEA.

[7] Magurran AE, McGill BJ (2011) Biological Diversity: Frontiers in Measuring Biodiversity. New York: Oxford University Press.

[8] Buckland ST, Studeny AC, Magurran AE, Illian JB, Newson JE (2011) The geometric mean of relative abundance indices: A biodiversity measure with a difference. Ecosphere 2: 100.

[9] Gotelli NJ, Colwell RK (2001) Quantifying biodiversity: Procedures and pitfalls in the measurement and comparison of species richness. Ecol Lett 4: 379–391.

[10] Gotelli NJ, Colwell RK (2011) Estimating species richness. In: Magurran AE, McGill BJ, editors. Frontiers in Measuring Biodiversity. New York: Oxford University Press. pp 39–54.

[11] Colwell RK, Chao A, Gotelli NJ, Lin SY, Mao CX, et al (2012) Models and estimators linking individual-based and sample-based rarefaction, extrapolation, and comparison of assemblages. J Plant Ecol 5: 3–21.

[12] Colwell RK (2011) EstimateS: Statistical estimation of species richness and shared species from samples, Version 9. Available: http://viceroy.eeb.uconn.edu/estimates. Accessed 2012 Sep 7.

[13] Thomas L, Buckland ST, Rexstad EA, Laake LJ, Strindberg S, et al (2010) Distance software: Design and analysis of distance sampling surveys for estimating population size. J Appl Ecol 47: 5–14. 20383262 10.1111/j.1365-2664.2009.01737.x PMC2847204

[14] Payton ME, Greenstone MH, Schenkerk N (2003) Overlapping confidence intervals or standard error intervals: What do they mean in terms of statistical significance? J Insect Sci 3: 34. 15841249 10.1093/jis/3.1.34 PMC524673

[15] Sokal RR, Rohlf FJ (1995) Biometry: The Principles and Practice of Statistics in Biology Research. New York: Freeman.

[16] SAS (2008) PC SAS Version 9.2.Cary, NC: SAS Institute.

[17] Limpert E, Stahel WA, Abbt M (2001) Log-normal distributions across the science: Keys and clues. BioScience 51: 341–352.

